# The role of CD36 in renal and bladder cancer

**DOI:** 10.15190/d.2026.4

**Published:** 2026-03-31

**Authors:** Mihai Ioan Pavalean, Victor Lucian Madan, Mihaela Cristina Pavalean, Laura Cristina Ceafalan, Mihail Eugen Hinescu

**Affiliations:** ^1^Faculty of Medicine, Carol Davila University of Medicine and Pharmacy, 050474 Bucharest, Romania; ^2^Emergency University Central Military Hospital, 010825 Bucharest, Romania; ^3^Victor Babeș National Institute of Pathology, 050096 Bucharest, Romania

**Keywords:** CD36, renal cell cancer, bladder cancer, lipid metabolism, metastasis, prognostic biomarker.

## Abstract

CD36 functions as both a lipid transporter and scavenger receptor, integrating metabolic and inflammatory signaling pathways. It plays a critical role in maintaining cellular homeostasis and influencing disease progression. This review summarizes the structure, ligands, functions, regulation, and clinical implications of CD36 in renal and bladder cancer. Increased CD36 expression promotes enhanced fatty acid uptake, which supports tumor cell proliferation, migration and survival, by mediating metabolic reprogramming and interacting with the tumor microenvironment. In renal cancer, most frequently clear cell renal carcinoma (ccRCC), which has a typical metabolic phenotype, CD36 is involved in lipid accumulation and oxidative stress pathways. Pathogenic mechanisms include hypoxia-inducible factor (HIF)-driven pathways and carnitine palmitoyl transferase 1A (CPT1A) via the PPARα/CD36 axis, which phosphorylate Akt. By using fatty acid oxidation, CD36 lead to the production of reactive oxygen species and to transcription of genes mediating a pro-tumor function, inducing tumor-associated macrophages (TAM). In bladder cancer, CD36 is implicated in tumoral cells proliferation, survival, and adaptation to metabolic stress, epithelial–mesenchymal transition (EMT) and influences the tumor microenvironment, through interactions with tumor-associated macrophages and inflammatory signaling pathways. Although multiple studies propose CD36 as a prognostic biomarker, inconsistencies across cohorts limit its clinical translation. Notably, advances have revealed the regulatory networks governing distinct physiological properties of CD36, thereby identifying targeting CD36 as a potential strategy for cancer treatment. Inhibition of CD36-mediated lipid metabolism and signaling pathways may reduce tumor growth and metastatic potential. However, further research is necessary to clarify its context-dependent functions and to develop effective CD36-targeted therapies. To our knowledge, this is the first review to systematically examine the role of CD36 across both renal and bladder cancer. It could be the first step toward identifying new mechanisms mediated by CD36 in these malignancies.

## SUMMARY

1. An overview of the CD36 receptor

2. The tissue distribution of the CD36 receptor in the urinary system and its functions

- 2.1. CD36 and the renal tissue

- 2.2. CD36 and the urinary bladder tissue

3. The UroOnco spectrum of CD36

- 3.1. Renal cell carcinoma

- 3.2. Urothelial (transitional cell) carcinoma

4. Potential prognostic markers for ccRCC

- 4.1. The role of CD36 in tumor progression and dissemination

- 4.2. The prognostic value of CD36

5. Conclusion

## 1. An overview of the CD36 receptor

CD36, also known as SR-B2, was first recognised as a receptor for thrombospondin (TSP). It is considered the archetypal class B scavenger receptor^1^. Scavenger receptors are currently categorized into ten classes (A–J) based on their sequence similarity or shared structural features. There is little to no sequence homology between different classes of scavenger receptors^2,3^. Mammalian class C scavenger receptors are currently not known, and the class C scavenger receptors have only been described in Drosophila melanogaster^2^*. *

From a structural standpoint, CD36 has an apparent molecular mass of 88 kDa due to extensive glycosylation^4^. It is a 472 amino acid protein and comprises of five distinct regions: the carboxy-terminal intracellular domain (COOH-terminal), the amino-terminal intracellular domain (NH2-terminal), an extracellular domain, and two transmembrane domains^5^. It contains two transmembrane domains and several palmitoylation sites that the C and N termini of CD36 are intracellular, and both ends are palmitoylated (3/7, 464/466). In addition to palmitoylation, the COOH terminus contains a set of ubiquitination sites (469/472)^6,7^ and has a motif of CXCX5K located on the cytosolic ends of the T cell co-receptors CD4 and CD8, which may be involved in the binding of src-related protein tyrosine kinases^8^.

**Figure 1:**
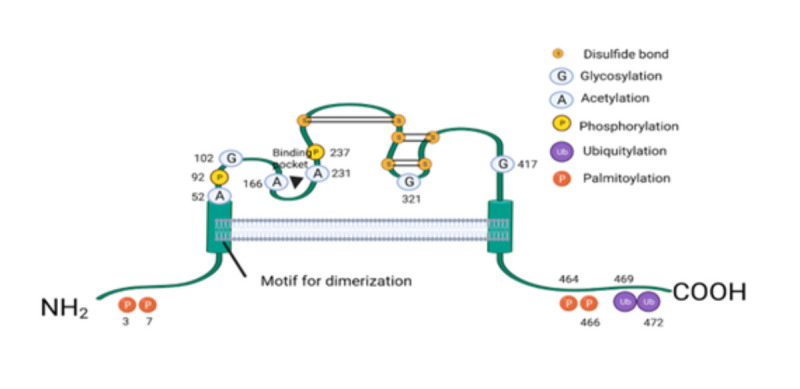
The structure of CD36 (Created in BioRender. Ceafalan, L. (2026) https://BioRender.com/zqoonwp)

The extracellular domains, recognized by the ligand, is a highly glycosylated hydrophobic ring containing ten glycosylation sites (79/102/134/163/205/220/ 235/247/321/417), two phosphorylation sites (92, 237), and three pairs of disulfide bonds (243-311/313-322/272-333)^6,9-11^. CD36 has the ability to bind numerous ligands, including advanced glycation end products (AGE) - a family of compounds that are the products of nonenzymatic reactions between reducing sugars and proteins, lipids, or nucleic acids, oxidized low density lipoprotein (oxLDL), fatty acids (FAs), collagen, TSP, and anionic phospholipids^12-16^. In the extracellular region of rat CD36, there are ten possible glycosylation sites, eight of which are conserved across humans and rats^17^ (Figure 1).

The previously described structure is essential for the recognition and endocytic uptake of oxidized phospholipids and modified low-density lipoprotein (LDL). Additionally, CD36 activity extends to the clearance of apoptotic cells, binding of amyloid proteins^18^, and involvement in inflammatory processes in atherosclerosis and Alzheimer's disease^19-22^. CD36 also acts as a potent endogenous inhibitor of angiogenesis, including tumor neovascularization^23^. In supporting this role, Hale et al. reported that deletion of CD36 in mice altered tumor growth and angiogenesis in the presence of thrombospondin type I repeat (TSR) proteins and demonstrated that Histidine-Rich Glycoprotein modulates this activity^23^.

## 2. The tissue distribution of the CD36 receptor in the urinary system and its functions

CD36 is expressed in microvascular endothelial cells (MVECs)^20^, stromal cells^24^, and immune cells^25^, and its level varies across these cell types. In malignant epidermal tumor cells, such as in the cells of ovarian cancer^26^, gastric cancer^27^, glioblastoma (GBM)^28^, and oral carcinoma^29,30^, CD36 expression is upregulated. In tumor microvessels, which support tumor development and metastasis, CD36 expression is generally downregulated^31^.

CD36 demonstrates a distinct and cell-type-specific distribution within the urinary system, indicating its involvement in renal lipid handling, tubular reabsorption, and local immune responses. In the kidney, CD36 is primarily expressed in proximal tubular epithelial cells, podocytes, mesangial cells, and endothelial cells, where it facilitates fatty acid uptake and lipid accumulation^6,32^. This distribution is functionally significant, as dysregulated CD36 expression has been linked to lipotoxicity, oxidative stress, and inflammation, which are key mechanisms in the development of renal disorders such as diabetic nephropathy and chronic kidney disease^6,31^.

Beyond the kidney, recent studies indicate that CD36 is expressed in additional components of the urinary tract, including the urothelium, where it may contribute to epithelial integrity and the regulation of inflammatory responses^29^. Alterations in CD36 expression and function have also been associated with tumorigenesis in urological malignancies, such as renal cell carcinoma and bladder cancer, highlighting its context-dependent biological significance^33,34^.

Due to its diverse functional roles and heterogeneous tissue distribution, characterizing the spatial expression patterns of CD36 within the urinary system is essential for clarifying its contributions to both normal physiology and disease.

### 2.1. CD36 and the renal tissue CD36

serves as the primary uptake system for free fatty acids in the kidney and is highly expressed in epithelial cells of the proximal and distal tubules, as well as in podocytes and mesangial cells (**Table 1**)^35,36^. Numerous functions of CD36 in renal tissue, healthy tissue distribution, and lipid metabolism have been described.

**Table 1:** CD36 ligands for every renal cell type and their effects Table caption

Cell type	CD36 ligand	Roles	Ref.
Proximal tubule epithelial cells	Fatty acids	ATP production, lipid accumulatio, apoptosis	^32,37^
	Albumin	Fibrosis	^35^
	AGEs	Apoptosis	^32^
	AOPPs	Inflammation, apoptosis	^37^
	Ox-LDL	Inflammation, apoptosis, ROS production	^38-41^
Monocytes and/or macrophages	Ox-LDL	Foam cell formation, ROS production, lipid accumulatio, apoptosis	^42^
Podocytes	Fatty acids	Lipid accumulatio, apoptosis, ROS production	^43-45^
Mesangial cells	Ox-HDL	Inflammation, apoptosis	^38^
Vascular endothelial cells	Fatty acids	ATP production	^46^
	Ox-LDL	Foam cell formation, lipid accumulatio, inflammation	^42^

CD36 functions as a critical upstream regulator of various inflammatory pathways in renal tubular epithelial cells, like NLR family pyrin domain-containing 3 (NLRP3) inflammasome activation by promoting mitochondrial reactive oxygen species (mtROS) generation. The NLRP3 inflammasome, a principal mediator of innate immune signalling, is increasingly recognized as a central contributor to inflammatory injury in diabetic nephropathy (DN)^43^. To demonstrate the mechanistic role of CD36 in diabetic nephropathy (DN), Hou et al. employed both *ex vivo* and *in vivo* (murine) models. They found that exposure to high glucose conditions induces significant metabolic reprogramming in renal tubular cells, characterized by a shift from oxidative phosphorylation (OXPHOS) to glycolysis, which promotes mtROS accumulation. Notably, CD36 exacerbates this process by inhibiting mitochondrial fatty acid oxidation (FAO), thereby reinforcing a feed-forward cycle of mitochondrial dysfunction and oxidative stress^44^.

Taken together, these findings establish CD36 as a central nexus of metabolic and inflammatory pathways connecting high glucose-induced metabolic stress to NLRP3 inflammasome activation. Consequently, targeting the CD36-mtROS-NLRP3 axis may represent a promising therapeutic strategy to mitigate inflammation-driven progression in DN.

Additionally, studies have demonstrated that increased dietary fat consumption, in the absence of circulating proprotein convertase subtilisin/kexin type 9, promotes renal lipid accumulation and subsequent renal injury^47^.

### 2.2. CD36 and the urinary bladder tissue

Although CD36 expression in bladder tissue is less extensively characterized than in renal structures, current evidence indicates that CD36 contributes to local lipid metabolism and innate immune surveillance. CD36 mediates the uptake of long-chain fatty acids, as a fatty acid translocase, into urothelial and stromal cells, thereby supporting membrane remodeling and cellular energy demands essential for maintaining urothelial barrier integrity and turnover^48,49^.

Beyond its metabolic functions, CD36 also acts as a pattern recognition receptor that participates in host defense mechanisms within the bladder. It binds a diverse array of endogenous and exogenous ligands, including oxidized lipids and microbial components, thereby facilitating the detection of pathogens and damaged cells. CD36 activates intracellular signaling pathways, such as NF-κB and MAPK, in cooperation with Toll-like receptors, which induce the production of pro-inflammatory cytokines and chemokines to coordinate the local immune response^50^.

Experimental evidence from urinary tract infection models indicates that CD36 plays a protective role in bladder immunity by promoting the clearance of bacteria and apoptotic neutrophils. Dysregulation of CD36 expression, including its downregulation by bacterial toxins, can impair pathogen clearance and intensify inflammation, leading to increased bacterial burden and tissue injury in the bladder^46^.

Collectively, these findings demonstrate that CD36 in the urinary bladder integrates metabolic and immune functions, contributing to epithelial homeostasis and the regulation of inflammatory responses during infection. This dual role emphasizes its significance as a key modulator of bladder physiology, especially under conditions of immune activation and tissue stress.

## 3. The UroOnco spectrum of CD36

Urological cancers, including renal and bladder malignancies, constitute a biologically heterogeneous group of tumors defined by intricate interactions among metabolic reprogramming, immune modulation, and microenvironmental adaptation. Renal cell carcinoma (RCC), originating in the renal cortex, accounts for eighty to eighty-five percent of primary renal cancers. Transitional cell carcinoma of the renal pelvis is the second most common, comprising approximately eight percent of cases. Other parenchymal epithelial tumors, such as oncocytomas, collecting duct tumors, and kidney sarcomas, are rare^51-53^. In the urinary tract, bladder cancer (BC) is the most prevalent and is increasingly recognized as a malignancy with significant metabolic reprogramming. Enhanced lipid uptake, storage, and β-oxidation, regulated in part by CD36 and PPAR signalling, contribute to tumor growth, survival, and therapeutic resistance^29^.

Recent studies have demonstrated an association between CD36 and tumor progression and metastasis in both RCC and BC. Inflammation and dysregulated lipid metabolism, mediated by CD36, are central to the pathogenesis of these cancers. Alterations in CD36 expression and function via signalling pathways have been implicated in cancer development, progression, and dissemination^32,33^.

### 3.1 Renal cell carcinoma

RCC has been classified into distinct subtypes:

1. Clear cell (seventy-five to eighty-five percent of tumors)-ccRCC,
2. Papillary (ten to fifteen percent),
3. Chromophobe (five to ten percent),
4. Oncocytic (three to seven percent),
5. Collecting duct (very rare),
6. Molecularly defined renal cell carcinomas (rare).

The most frequent histological subtype of renal cancer, clear cell renal cell carcinoma (ccRCC), is characterized by the accumulation of intracellular lipids and glycogen. This distinctive metabolic phenotype reflects alterations in lipid metabolism, which are now recognized as a hallmark of ccRCC biology.

To better understand the processes underlying CD36-mediated ccRCC development, several mechanisms have been described in studies. One new theoretical explanation for the pathogenesis of ccRCC is the regulatory role of carnitine palmitoyl transferase 1A (CPT1A) via the PPARα/CD36 axis. CPT1A is located on the outer mitochondrial membrane and is a rate-limiting fatty acid oxidation (FAO) enzyme that transports fatty acids into mitochondria for oxidation by converting acyl-CoA into acyl-carnitines. CPT1A expression is crucial for lipid accumulation, which promotes ccRCC development. Therefore, CPT1A prevents cholesterol uptake and lipid accumulation by increasing peroxisome proliferator-activated receptor α (PPARα) levels through regulation of CD36, with further control of Akt phosphorylation implicated in ccRCC growth. Also, Akt activation, which regulates cell survival under stress conditions in RCC, is linked to cancer survival and proliferation^57^.

The accumulation and storage of lipids are critical for the management of oxidative and endoplasmic reticulum (ER) stress, and lipids promote metastasis in ccRCC tumors^58-60^.

Another explanation for the pathogenesis of ccRC is the activation of the hypoxia-inducible factor (HIF) signaling pathways, which remains poorly understood. Hypoxic conditions further enhance CD36-mediated lipid remodeling. HIF1α signaling increases the expression of lipid receptors, such as CD36 and ACVRL1, as well as genes involved in lipid transport (FABP7) and storage (PLIN2, HILPDA), thereby reinforcing a lipid-rich tumor phenotype^61^.

Additionally, Liao et al. demonstrated that hypoxia directly induces CD36 expression through HIF-2α activation. Functional studies show that CD36 knockdown reduces lipid accumulation and eliminates HIF-2α-driven tumor-promoting effects, establishing CD36 as a key mediator of hypoxia-induced metabolic reprogramming^62,63^.

Moreover, since CD36 is expressed not only in renal tumor cells but also in macrophages, some studies have focused on the tumor microenvironment (TME), suggesting potential links between ccRCC lipid metabolism and its immune infiltrate, particularly tumor-associated macrophages (TAM). Therefore, CD36 plays an important role in fatty acid transport, macrophage lipid accumulation, and immune modulation^64^. In a murine model, Su et al. reported that CD36 plays a key role in TAMs for lipid accumulation and tumor development. It showed that TAMs express CD36 and use fatty acid oxidation, leading to production of reactive oxygen species and to transcription of genes mediating a pro-tumor function (M2-like phenotype)^65^.

This CD36-mediated influence on macrophage polarization correlates with immunosuppression, thereby reducing the effectiveness of anti-tumor immunity in the kidney tumour microenvironment^64^.

In summary, these findings establish CD36 as a critical convergence point that integrates hypoxia signalling and lipid metabolism in ccRCC. By interacting with HIF-driven pathways and PI3K/Akt signalling, CD36 maintains a lipid-dependent oncogenic state, highlighting its potential as a therapeutic target in metabolically reprogrammed renal tumors.

### 3.2 Urothelial (transitional cell) carcinoma

Bladder cancer is the most prevalent neoplasm of the urinary tract and is classified into two types with distinct molecular characteristics. In 75% of cases, the disease is confined to the mucosa and is termed non-muscle-invasive bladder cancer (NMIBC). The remaining cases are classified as muscle-invasive bladder cancer (MIBC)^66^.

CD36-mediated fatty acid transport improves the availability of exogenous lipids, which tumor cells use to support proliferation, survival, and adaptation to metabolic stress. This lipid dependency is especially significant in bladder cancer, where changes in lipid metabolism are increasingly recognized as a hallmark of tumor progression^67^.

CD36 also influences the tumor microenvironment and contributes to aggressive disease behaviour. Clinical studies indicate that CD36 expression is associated with advanced pathological stages in MIBC, supporting its role in tumor progression and dissemination^68^. NMIBC tumor samples exhibit increased expression of the fatty acid transporters FATP4, CD36, and ACSL1^69^.

Regarding molecular changes in BC after radiotherapy, Shang et al. sought to identify the mechanisms and signal transduction pathways. They investigated the profile of messenger RNA (mRNA) and long non-coding RNA (lncRNA). The expression of matrix metalloproteinase MMP-3, MMP-10, MMP-12, and MMP-13 was significantly increased in BC after RTx, whereas the expression of CD36 was decreased^70^.

We also perform a study that examines a panel of 26 dysregulated microRNAs (miRNAs) in BC, several of which may be associated with tumor aggressiveness and increased risk of disease progression^71^.

Collectively, current evidence demonstrates that CD36 plays multiple roles in bladder cancer by promoting lipid-driven metabolic reprogramming, sustaining cancer stemness, and facilitating tumor progression.

## 4. Potential prognostic markers for ccRCC

Identifying molecular markers linked to poor prognosis in renal cell carcinoma is crucial for elucidating tumor heterogeneity and informing personalized therapeutic strategies (Table 2).

**Table 2:** Markers that are potentially associated with a worse prognosis for ccRCC

Markers	Ref.
Human B7 homolog 1 (B7H1) and 4 (B7H4) expression	^72^
Low levels of carbonic anhydrase IX (CAIX)	^73^
High levels of the proliferation marker Ki-67	^73^
Higher levels of hypoxia-inducible factor (HIF)-1 alpha expression	^74^
Expression of the U3 small nucleolar ribonucleoprotein (IMP3)	^69,75,76^
Deletion of chromosome 9p	^77^
Mutations of tumor suppressor genes on chromosome 3p21, including mutations of breast cancer type 1 (BRCA1)-associated protein 1 (BAP1) and SET domain containing 2 (SETD2)	^78^
PD-L1/PD-1	^79^

We found evidence for a continuous crosstalk between these markers and CD36 in other malignant pathologies. Co-expression of PD-L1/PD-1 with CXCR3/CD36 in circulating lymphocytes and serum IL-19 levels explain poor prognosis and can be considered potential markers for extranodal involvement in lymphoma. Co-expression was evaluated by flow cytometry in seventy-eight lymphoma patients before and after therapy. There also was 50 healthy controls in the study^79^.

The relationship between Ki67 and CD36 was demonstrated in oral squamous cell carcinoma. CD36-positive cells demonstrated increased expression of Ki-67 and migration activity compared with CD36**-**negative cells^80^.

Hypoxia and hypoxia-inducible factors (HIFs) play essential and multiple roles in renal ischemia-reperfusion injury (IRI). Qu et al. demonstrated a new role for the HIF-2α/CD36 regulatory axis in rewiring dendritic cell (DC) lipid metabolism under IRI-associated hypoxia. This is a potential therapeutic target to resolve long-standing obstacles in the treatment of this severe complication^81^.

### 4.1 The role of CD36 in tumor progression and dissemination

In bladder cancer, elevated CD36 expression is linked to increased tumor growth, invasiveness, and the development of aggressive phenotypes. CD36 has been known to be associated with altered lipid metabolism and initiation of metastasis, contributing to cancer progression^29,67,75^. High expression of FATP4, CD36, and long-chain acyl-CoA synthetase 1 (ACSL1) has been associated with metastasis. Patients with NMIBC who demonstrate high expression of these fatty acid transporters need to be closely monitored and treated more aggressively^69^. Additionally, CD36 is implicated in the maintenance of cancer stem cell populations, which are essential for tumor initiation, therapy resistance, and metastatic spread^82^.

Overexpression of CD36in cell lines or patient-derived cells with low metastatic potential greatly increased their potential to metastasize to lymph nodes, with penetrance increasing from less than 20% to 75-80%^29^. The link between CD36 and the initiation of the metastatic process was also demonstrated, without being able to specify exactly how this occurs, because CD36+ cells are not only capable of initiating metastasis but can also recapitulate their molecular and cellular heterogeneity from the primary origin^29^. Clinical evidence further supports this role, as elevated CD36 expression correlates with advanced tumor stage and poorer prognosis in bladder cancer patients^68^.

Current evidence regarding the role of CD36 investigates the molecular mechanism and signal transduction pathways implicated in BC after Rtx. They analysed the mRNA and lncRNA profile. One of the study’s conclusions was that several genes were down-regulated in BC after RTx, including CD36^70^. Additionally, endostatin gene therapy in RCC can also have a role in treatment, reducing the number of lung tumor nodules, reducing metastasis and resulting in a higher survival rate^76,83^.

Collectively, these findings support a role for CD36 in the regulation of metastatic processes, both through tumor-intrinsic mechanisms and via modulation of the tumor microenvironment.

### 4.2. The prognostic value of CD36

In several types of cancers, there is a positive correlation between CD36 expression in tumor cells and poor clinical outcome, as illustrated in Table 3.

**Table 3:** The role of CD36 in various types of cancers

Type of cancer	Role of CD36	Ref.
Oral squamous cell carcinoma	CD36 depletion greatly reduced the size of lymph node metastases in all tumor lines	^29^
Human melanoma	Impairs metastasis – inhibition of CD36	^29^
Breast cancer	Impairs metastasis – inhibition of CD36	^29,84^
Cervical cancer	Poor tumor differentiation, lymph node metastasis	^85^
Gastric cancer	Contribute to the tumor proliferation	^27,86^
Bladder cancer	Promots bladder cancer	^45^
Prostate cancer	Associated with reduced relapse-free survival and increased incidence in metastases	^87^
Cholangiocarcinoma	Cancer relapse	^88^
Hepatocellular carcinoma	CD36 knockdown - impairs cell proliferation, decreases migration and invasion	^82^
Colorectal cancer	Associated with metastatic tumors, reduced 5-year survival	^89^
Leukemias and lymphomas	CD36 knockdown - decreases FA uptake and increases chemotherapeutic-induced apoptosis	^90^
Glioblastoma	Elevated capacity to form tumorspheres in culture	^82^

The CD36 receptor has gained increasing attention as a prognostic biomarker in urological malignancies, including both bladder cancer and renal cell carcinoma, due to its involvement in lipid metabolism, tumor progression and immune regulation.

Although direct clinical data remain limited in RCC, emerging evidence indicates that CD36 expression correlates with increased tumor aggressiveness and unfavourable clinical outcomes. We identified 2 studies with CD36 expression that support the above, one of which demonstrated a correlation between mRNA responsible for the CD36 cluster and visceral adipose tissue (VAT), based on the premise that a higher VAT rate leads to a more aggressive tumor progression or a worse prognosis for patients with RCC^91^ and the other suggest that the transport of FA through the cell membrane, facilitated by CD36 and FATP4, could be pathognomonic for RCC. Also, it demonstrated a strong correlation between oncogenesis and tumor progression and the simultaneous overexpression of both FATP4 and CD36^92^.

In addition to protein-level alterations, transcriptomic analyses have identified regulatory RNA networks associated with RCC prognosis. Seven mRNA targets of miRNA-21 and 12 mRNA targets of miRNA-155 were identified as key prognostic factors for patients with RCC. These two miRNAs were significantly related to 5-year survival (*p* < 0.05). The analysis of the competitive endogenous RNA (ceRNA) regulatory network provided new and useful perspectives for the development of further therapeutic strategies for RCC^93^.

Clinical studies in bladder cancer have shown that CD36 expression correlates with increased tumor aggressiveness and poorer clinical outcomes. For instance, immunohistochemical analyses in muscle-invasive bladder cancer revealed that CD36-positive tumors correlate with advanced pathological stage and a trend toward lymph node involvement. Importantly, patients with CD36-positive tumors exhibited significantly shorter disease-free survival compared to those with CD36-negative tumors, supporting its role as a negative prognostic marker^68^.

Furthermore, alterations in lipid transport pathways involving CD36 are associated with tumor progression and increased metastatic potential in bladder cancer. Elevated expression of CD36 and other fatty acid transporters enhances lipid uptake, thereby promoting tumor growth, invasion, and resistance to therapy. These findings further underscore the prognostic significance of CD36^69^. It was also demonstrated that the fatty acid uptake by tumor cells increased, and the fatty acid was depleted within the tumor microenvironment through the interaction of circZNF609 with IGF2BP2 and CD36^94^.

Additionally, there is a connection between time to progression (TTP) and individual gene expression, adjusting for clinical covariates, in a specific type of bladder cancer. The study refers to a high-risk non-muscle-invasive (HGT1) micropapillary variant of bladder cancer (MPBC). Furthermore, high expression of FABP3 and CD36 was associated with shorter TTP^95^. Collectively, these findings indicate that CD36 represents a promising prognostic biomarker in both BC and RCC, reflecting its central role in lipid-driven tumor progression and immune regulation. Its expression may help stratify patients by risk and could serve as a potential therapeutic target in the future.

## 5. Conclusion

CD36 has recently been identified as a promising therapeutic target across multiple cancer types, offering significant clinical implications. In kidney and bladder cancer, high levels of CD36 could be considered a marker for poor prognosis. Even if the mechanisms underlying CD36's role in oncogenesis remain poorly understood, CD36 inhibitors could be considered for cancer therapy in the near future. Targeting CD36 may offer therapeutic benefit, but given its systemic metabolic roles, strategies such as tumor-specific delivery or pathway-selective inhibition will be essential. Further clinical trials are needed to investigate the regulation and downstream signalling pathways of CD36 during the manipulation of the target in precise clinical translation. Although evidence increasingly supports the role of CD36, significant gaps persist in understanding the molecular pathways that regulate its activity and its interactions with stromal and immune components. Recognising CD36 at the intersection of metabolic regulation and tumor ecology underscores its potential as both a biomarker and a therapeutic target.
